# C1 Domain-Targeted Isophthalate Derivatives Induce Cell Elongation and Cell Cycle Arrest in HeLa Cells

**DOI:** 10.1371/journal.pone.0020053

**Published:** 2011-05-23

**Authors:** Virpi Talman, Raimo K. Tuominen, Gustav Boije af Gennäs, Jari Yli-Kauhaluoma, Elina Ekokoski

**Affiliations:** 1 Division of Pharmacology and Toxicology, Faculty of Pharmacy, University of Helsinki, Helsinki, Finland; 2 Division of Pharmaceutical Chemistry, Faculty of Pharmacy, University of Helsinki, Helsinki, Finland; Cornell University, United States of America

## Abstract

Diacylglycerol (DAG)-mediated signaling pathways, such as those mediated by protein kinase C (PKC), are central in regulating cell proliferation and apoptosis. DAG-responsive C1 domains are therefore considered attractive drug targets. Our group has designed a novel class of compounds targeted to the DAG binding site within the C1 domain of PKC. We have previously shown that these 5-(hydroxymethyl)isophthalates modulate PKC activation in living cells. In this study we investigated their effects on HeLa human cervical cancer cell viability and proliferation by using standard cytotoxicity tests and an automated imaging platform with machine vision technology. Cellular effects and their mechanisms were further characterized with the most potent compound, HMI-1a3. Isophthalate derivatives with high affinity to the PKC C1 domain exhibited antiproliferative and non-necrotic cytotoxic effects on HeLa cells. The anti-proliferative effect was irreversible and accompanied by cell elongation. HMI-1a3 induced down-regulation of retinoblastoma protein and cyclins A, B1, D1, and E. Effects of isophthalates on cell morphology, cell proliferation and expression of cell cycle-related proteins were different from those induced by phorbol 12-myristate-13-acetate (PMA) or bryostatin 1, but correlated closely to binding affinities. Therefore, the results strongly indicate that the effect is C1 domain-mediated.

## Introduction

The protein kinase C (PKC) family of serine/threonine kinases consists of ten known isozymes that can be divided into three classes based on their regulatory domain structure and activation properties [1]. The classical PKCs (α, βI, βII and γ) and novel PKCs (δ, ε, η and θ) contain a duplicated diacylglycerol (DAG) and phorbol-responsive C1 domain, whereas the single C1 domain of atypical PKCs (ζ and ι/λ) does not respond to DAG or phorbol esters. PKC C1 domains are cysteine-rich sequences that are approximately 50 amino acids long and are located within the regulatory region of the enzyme. They are folded into zinc finger-like structures that respond to increased DAG levels at the plasma membrane, leading to relocation and activation of PKC isoforms. DAG is generated by phospholipase C-mediated hydrolysis of phosphatidylinositol-4,5-bisphosphate (PIP2) after activation of G protein-coupled receptors or receptor tyrosine kinases [2]. Alternatively, it can be produced indirectly from phosphatidylcholine via phospholipase D and phosphatic acid phosphatase [2]. Phorbol esters are diterpene-structured natural compounds that mimic DAG actions but with significantly higher potency [3].

PKC is implicated in the regulation of various cellular functions, such as differentiation, proliferation, apoptosis, motility and malignant transformation [4]. However, substrates and physiological roles of individual isozymes are still mostly unknown. PKCs have been the subject of intensive research and drug development since the discovery that they are receptors for tumor-promoting phorbol esters. Specifically, PKC is considered a potential cancer drug target due to its role in cell proliferation and apoptosis. This hypothesis has been strengthened by findings of abnormal PKC expression levels and/or aberrant PKC activity in various cancer types [5]. In addition to cancer, PKC plays a role in several other diseases, including cardiovascular diseases, diabetic complications, and Alzheimer's disease [6]–[8].

C1 domains of classical and novel PKC isozymes represent the first recognized effectors for the physiological second messenger DAG and tumor-promoting phorbol esters [1], [2], and for a long time PKCs were considered the only phorbol ester effectors. However, DAG/phorbol-responsive C1 domains have been found in the following six other classes of proteins: (1) protein kinase D (PKD) family; (2) DAG kinases (DGKs); (3) Ras guanyl nucleotide-releasing proteins (RasGRPs); (4) chimaerins; (5) Munc13 scaffolding proteins; and (6) myotonic dystrophy kinase-related Cdc42-binding kinases (MRCKs) [9], [10]. Although physiological roles of non-PKC phorbol ester receptors have been only partially elucidated, signaling pathways regulated by these proteins are central in controlling various cellular functions thereby affecting many pathological conditions. Particularly, several C1 domain-containing proteins participate in regulating cell proliferation, apoptosis and/or motility, thus these proteins might be implicated in cancer [4].

The C1 domain is regarded as an attractive drug target because PKCs and other DAG-responsive C1 domain-containing proteins play key roles in controlling cell proliferation, apoptosis, and motility [11]. Various C1 domain ligands have been described, including some obtained from natural sources and others based on synthesized chemical entities (reviewed in [12]). Several C1 domain-binding compounds, such as the natural compounds bryostatin 1 and ingenol-3-angelate (PEP005), have entered clinical trials for the treatment of different cancers [13]. However, most C1 domain ligands are structurally complex so modification and large-scale production may be unfeasible. Our group has developed a novel class of synthetic C1 domain ligands, dialkyl *5-*(hydroxymethyl)isophthalates, which are fairly easily synthesized with good yields, bind to the phorbol ester binding site within the PKC C1 domain, and modulate PKC activity in living cells [14]. The purpose of this study was to examine effects of nine isophthalate derivatives on HeLa human cervical cancer cell viability and proliferation and to further characterize cellular effects of one of the active derivatives.

## Results

### Compound selection

**Figure 1:**
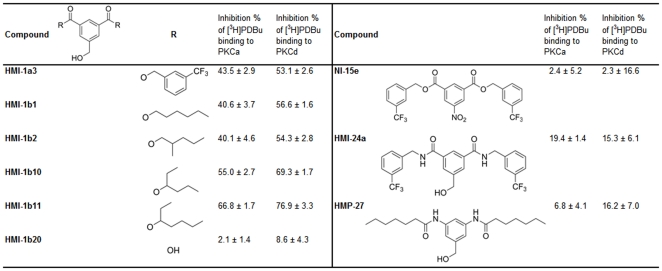
Structures and binding affinities of isophthalate derivatives. Binding affinity is expressed as mean ± SEM (n = 3–8) of the inhibition percentage of [^3^H]phorbol-12,13-dibutyrate ([^3^H]PDBu) binding at compound concentrations of 20 µM. Binding data are from [14] and are reprinted with permission from the American Chemical Society.

To evaluate effects of isophthalate derivatives on HeLa cell viability and proliferation, we selected compounds based on our previous studies with over 40 compounds [14]. Five compounds were selected for their high binding affinity to the C1 domain (“active” compounds; HMI-1a3, HMI-1b1, HMI-1b2, HMI-1b10, and HMI-1b11) and four compounds were selected that had poor binding affinity (“inactive” compounds; HMI-1b20, NI-15e, HMI-24a, and HMP-27). Compound structures and their binding affinities to PKCα and PKCδ are shown in Figure 1. Additionally, the well-characterized C1 domain ligands phorbol 12-myristate-13-acetate (PMA) and bryostatin 1 were used as reference compounds.

### Effects of isophthalates on HeLa cell viability

**Figure 2:**
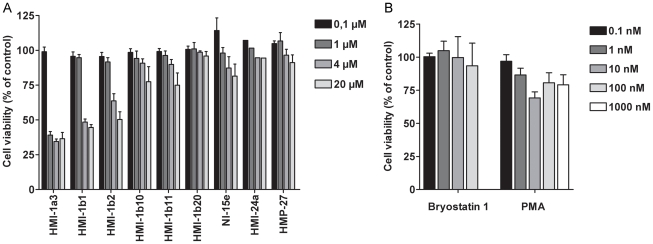
Effects of C1 domain ligands on HeLa cell viability. Cells were exposed to isophthalate derivatives (A) and PMA or bryostatin 1 (B) for 24 h, and cell viability was determined by MTT assay. Results are expressed as mean + SEM (n = 3–6; n = 2 for the HMI-24a group).

HeLa cells were exposed to isophthalate derivatives for 24 or 48 h, and cell viability was studied using standard LDH and MTT tests. A 24-h exposure to HMI-1a3 or HMI-1b1 at concentrations of ≥1 µM or ≥4 µM, respectively, induced cell viability reductions that were greater than 50% (Fig. 2A, p<0.001). In addition, compounds HMI-1b2 (4–20 µM, p<0.001), HMI-1b11 (20 µM, p<0.01), and HMI-1b10 (20 µM, p<0.05) induced statistically significant cytotoxicity after a 24-h treatment (Fig. 2A). No significant toxicity was observed after exposure to compounds HMI-1b20, NI-15e, HMI-24a, and HMP-27 (Fig. 2A). MTT tests after a 48-h treatment gave similar results (data not shown). In contrast, bryostatin 1 had no significant effect on HeLa cell viability (p = 0.964; Fig. 2B). PMA exhibited only weak toxicity; it induced a 30% reduction in cell viability at 10 nM (p<0.01) and 20% reductions in viability at 0.1–1 µM that were however not statistically significant (Fig. 2B). In contrast to MTT assay results, LDH tests showed no significant cytotoxicity with any compound. Specifically, cytotoxicity measured by the LDH test was always less than 10% (data not shown).

### Inhibition of thymidine incorporation by HMI-1a3

**Figure 3:**
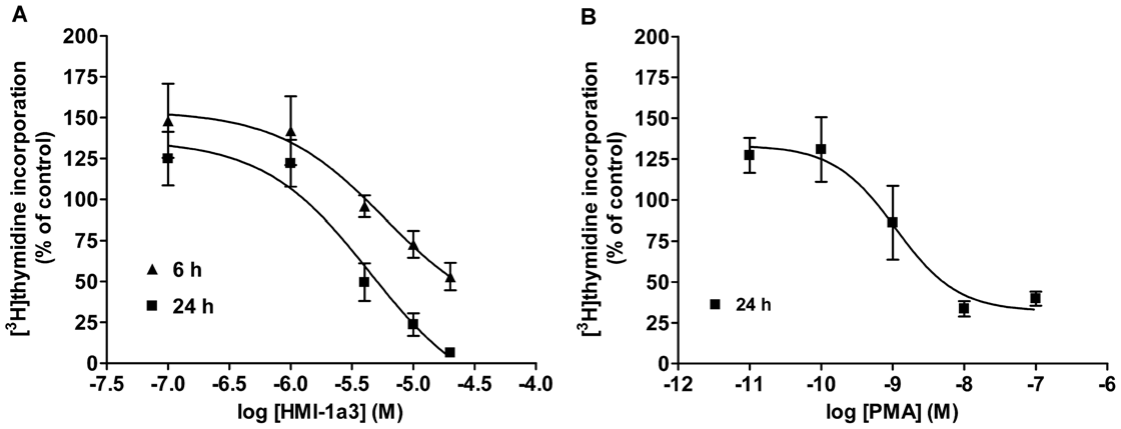
Inhibition of [^3^H-methyl]thymidine incorporation by HMI-1a3 and PMA. HeLa cells were treated for 6 or 24 h with increasing concentrations of HMI-1a3 (A) or PMA (B), and the incorporation of thymidine was determined. Error bars indicate SEM from 3 independent experiments.

On the basis of the cell viability studies we chose the most potent compound HMI-1a3 for studying its effects on thymidine incorporation. Exposures to HMI-1a3 for 6 and 24 h inhibited the incorporation of [methyl-^3^H]thymidine into HeLa cells in a concentration-dependent manner (Fig. 3A). The EC_50_ values for HMI-1a3 were 5.8 µM and 4.5 µM for the 6 and 24 h incubations, respectively. However, HMI-1a3 concentrations of 0.1 µM and 1 µM increased thymidine incorporation by 25–50% after both exposure durations. PMA inhibited thymidine incorporation with an EC_50_ value of 1.9 nM after a 24-h treatment (Fig. 3B). Similar to HMI-1a3, the smallest PMA concentrations of 0.01 nM and 0.1 nM induced a 25–30% increase in thymidine incorporation.

### Effects of isophthalates on HeLa cell proliferation

**Figure 4:**
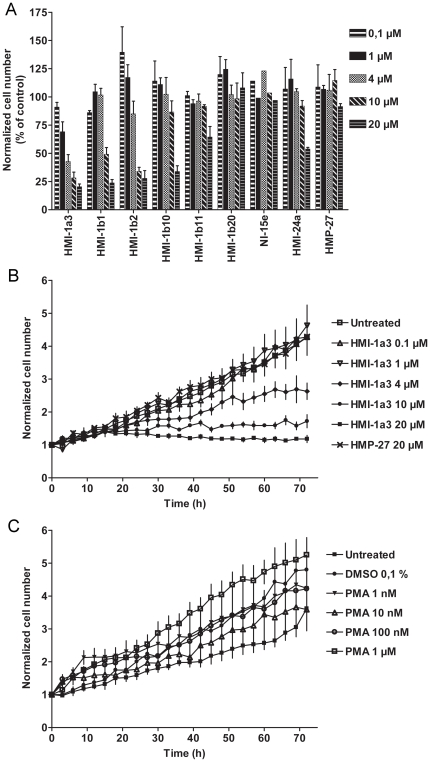
Effects of isophthalate derivatives and PMA on HeLa cell proliferation. HeLa cells were treated with test compounds and photographed automatically for 72 h with Cell-IQ®. Cell numbers were quantified from each image using Cell-IQ Analyzer® software. A, normalized total cell number at 72 h is expressed as a percentage of control. Results represent mean + SEM from 3–5 independent experiments except for NI-15e where n = 2. B and C, proliferation of HeLa cells during the 72-h experiment is shown as normalized total cell number in untreated wells and after treatment with different concentrations of HMI-1a3 and HMP-27 (B) and PMA (C). Cell numbers shown were determined from images taken with 3-h intervals from a single, representative experiment. Error bars correspond to SEM of 4 images taken from different positions within the same well. Experiments were repeated at least 3 times with similar results.

A continuous cell culturing platform with integrated phase contrast optics (Cell-IQ®) was used to study the effects of isophthalates on HeLa cell proliferation in more detail. Active compounds inhibited cell proliferation in a concentration-dependent manner (Fig. 4A and 4B). After a 72-h treatment, statistically significant inhibition was measured for the following compounds compared to untreated HeLa cells (Fig. 4A): HMI-1a3 at 4–20 µM (p<0.001); HMI-1b1 and HMI-1b2 at 10–20 µM (p<0.001 and p<0.01, respectively); and HMI-1b11, HMI-1b10 and HMI-24a at 20 µM (p<0.05). Compounds HMI-1b20, NI-15e and HMP-27 had no effect on HeLa cell proliferation (Fig. 4A). The concentration-dependent effect of HMI-1a3 was detectable for the duration of the experiment (Fig. 4B), and this pattern was also detected among other compounds that inhibited cell proliferation (data not shown). The proliferation rate of HeLa cells exposed to inactive compounds did not differ from untreated cells at any time point (HMP-27 shown as an example in Fig. 4B). Despite the fact that PMA inhibited thymidine incorporation in HeLa cells, it had no anti-proliferative effect in HeLa cells in Cell-IQ® experiments (Fig. 4C).

### Effects of isophthalates and PMA on HeLa cell morphology

**Figure 5:**
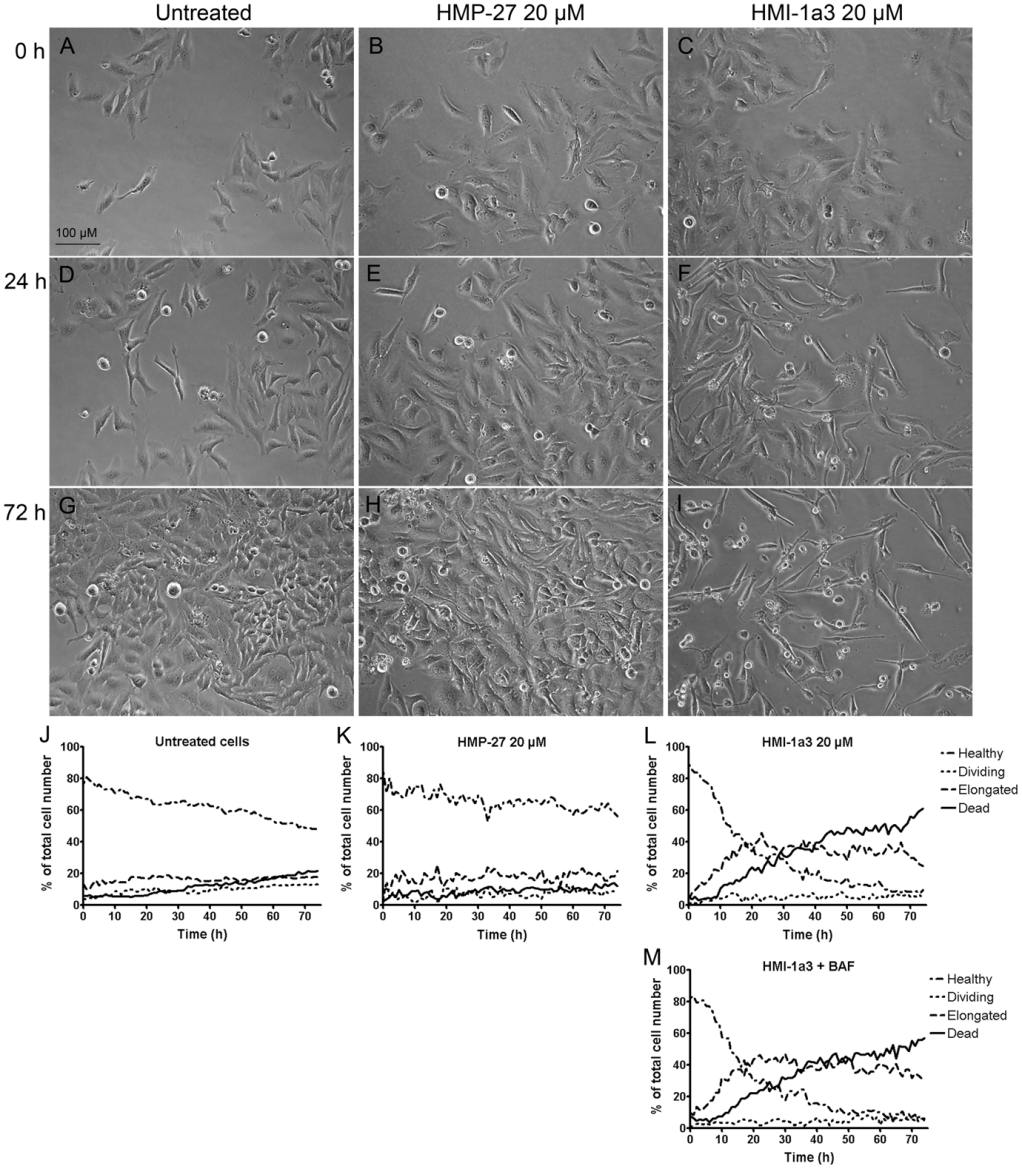
Effect of isophthalate derivatives on HeLa cell morphology. Untreated cells (A, D, G) and cells treated with 20 µM of HMP-27 (B, E, H) or 20 µM of HMI-1a3 (C, F, I) were imaged automatically for 72 h with Cell-IQ® and analyzed using Cell-IQ Analyzer® software. Representative photomicrographs taken at time points 0 h (A–C), 24 h (D–F) and 72 h (G–I) are presented. J–M, quantification results from a single, representative experiment (mean from 4 images in different positions within the same well) of untreated cells (J) and cells treated with 20 µM of HMP-27 (K), 20 µM of HMI-1a3 (L) or 20 µM of HMI-1a3 and 40 µM of BAF. Experiments were repeated at least 3 times with similar results.

Active isophthalates, such as HMI-1a3, induced changes in HeLa cell morphology that were characterized by cell elongation and reduced cell-to-cell contacts (Fig. 5), while inactive compounds had no effect (HMP-27 shown in Fig. 5). This observation led us to develop a protocol for Cell-IQ Analyzer® software for quantifying the morphological changes (see experimental section for details). In untreated wells and inactive compound-treated wells (e.g., HMP-27) the proportion of apparently healthy cells remained the same or decreased slightly and the proportion of dividing and dead cells increased only slightly during the 72-h exposure (Fig. 5, J–K). However, the proportion of apparently healthy cells decreased immediately and rapidly in wells treated with 20 µM of HMI-1a3 (Fig. 5L). This decline was accompanied with an increase in the proportion of elongated cells and a delayed increase in the proportion of dead cells. Elongated cells constituted the majority after 22 h, and dead cells dominated wells after 40 h of exposure. At 10 µM concentration HMI-1a3 induced similar changes in cell morphology, although at a slower rate (data not shown). In addition, the proportion of dead cells never exceeded the proportion of elongated cells in wells treated with 10 µM of HMI-1a3. Other active compounds (HMI-1b1, HMI-1b2, HMI-1b10, and HMI-1b11) also induced cell elongation at concentrations that inhibited cell proliferation (data not shown). However, compound HMI-24a, which inhibited proliferation of HeLa cells to some extent at the highest concentration (20 µM) (Fig. 4A), induced only slight changes in cell morphology (data not shown). HMI-1a3-induced cell elongation and cell death were not affected by co-exposure to the pan-caspase inhibitor Boc-Asp(OMe) fluoromethyl ketone (BAF; Fig. 5M).

**Figure 6:**
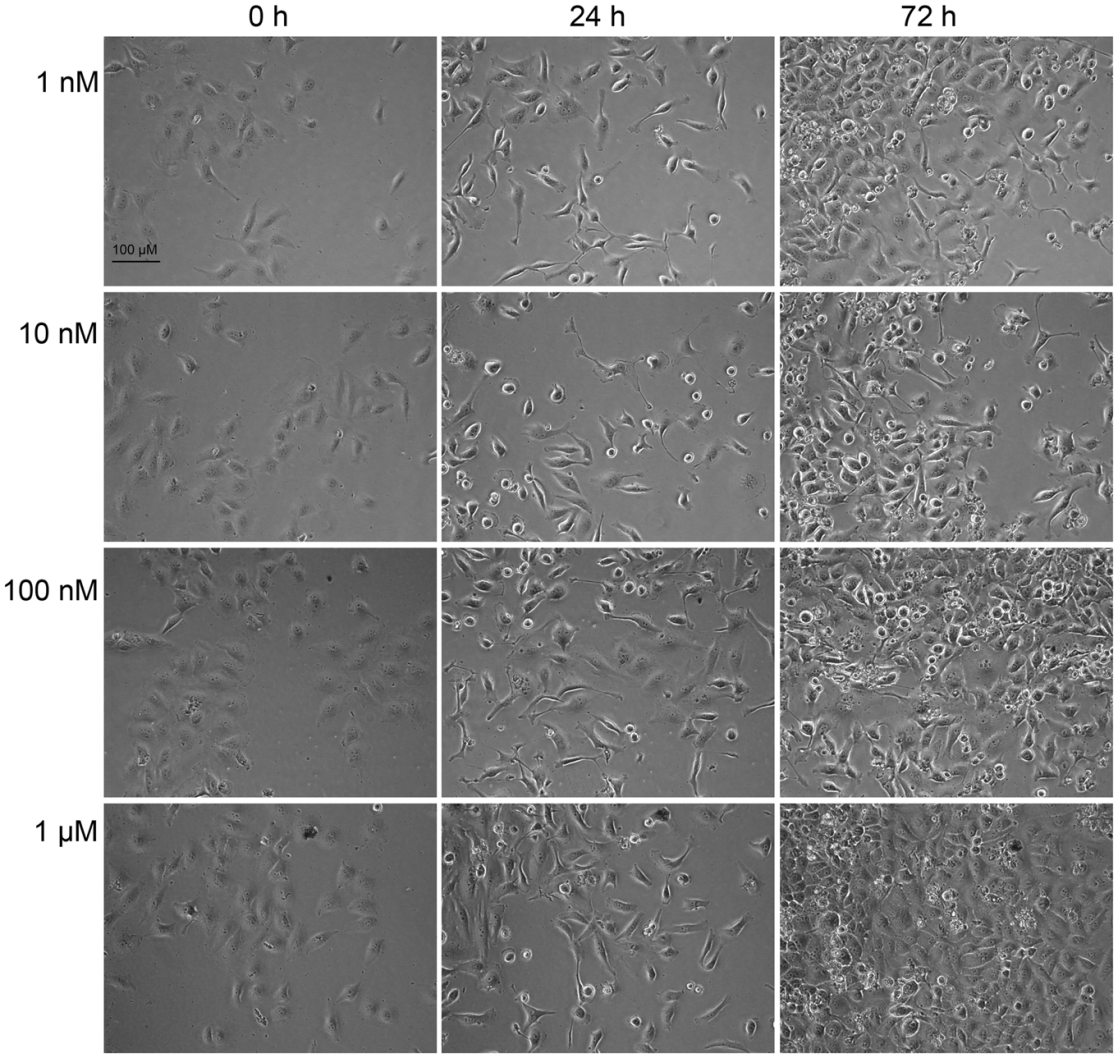
Effect of PMA on HeLa cell proliferation and morphology. HeLa cells treated with PMA (1 nM–1 µM) were imaged for 72 h with Cell-IQ®. Representative photomicrographs taken at time points 0, 24 and 72 h are presented. Experiments were repeated 3 times with similar results.

PMA induced a different pattern of morphological changes in HeLa cells compared to isophthalate-induced changes. More specifically, it evoked a transient elongation that was visible at 12–30 h after the beginning of the experiments, as well as cell rounding that was persistent throughout the experiments (Fig. 6). In contrast to isophthalate-treated cells, in which elongation was accompanied with permanent growth arrest, cells that were elongated after PMA treatment recovered and continued dividing (Fig. 6).

### The effects of HMI-1a3 on HeLa cell proliferation and morphology are irreversible

To determine whether cellular effects of HMI-1a3 were reversible, HeLa cells were treated with 20 µM of HMI-1a3 for 1, 8 or 24 h, washed twice with PBS, and grown in fresh medium for an additional 72 h (total duration of experiment was 96 h). Untreated cells and cells treated with HMI-1a3 for the duration of the experiment were used as controls. Cells were inspected daily with a microscope, and cell morphology and proliferation were assessed visually. After a 24-h treatment with 20 µM of HMI-1a3 followed by a 72-h incubation period in normal medium, cell morphology and proliferation rate were identical to those treated with 20 µM of HMI-1a3 for the duration of the experiment, i.e. most cells were elongated and unable to divide or were dead (data not shown). In wells exposed to HMI-1a3 for 8 h, only a fraction of cells was elongated and unable to divide, while the remainder of cells continued to proliferate. At the end of the experiment, there were fewer cells than in untreated wells, but considerably more cells than in wells treated with HMI-1a3 for 24 or 96 h (data not shown). A one-hour exposure to 20 µM of HMI-1a3 induced only slight reductions in cell density and few cell elongations with most cells appearing normal and healthy (data not shown).

### HMI-1a3-induced changes in the expression of cell cycle markers are different from those induced by PMA or bryostatin 1

**Figure 7:**
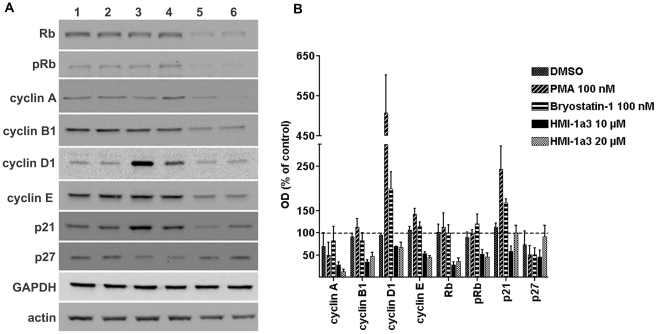
Effects of C1 domain ligands on the expression of cell cycle markers. HeLa cells were exposed to compounds for 24 h and proteins were detected with Western blotting. A, representative Western blots showing specific immunoreactive bands after various treatments (lanes 1–6) as follows: lane 1, untreated cells; lane 2, 0.2% DMSO; lane 3, PMA at 100 nM; lane 4, bryostatin 1 at 100 nM; lane 5, HMI-1a3 at 10 µM; lane 6, HMI-1a3 at 20 µM. B, quantification results of Western blots expressed as mean + SEM (n = 3-4). OD = optical density.

Effects of a 24-h treatment with HMI-1a3 on the expression of cell cycle markers were studied and compared to those of PMA and bryostatin 1. Representative blots are shown in Fig. 7A and the quantification results are provided in Fig. 7B. At HMI-1a3 concentrations of 10 µM and 20 µM, down-regulation was observed among all cyclins tested (i.e., cyclin A, cyclin B1, cyclin D1, and cyclin E), and decreases in amounts of retinoblastoma (Rb) protein and phosphorylated Rb (pRb) were detected. Additionally, 10 µM of HMI-1a3 down-regulated p21^waf1/cip1^ and p27^kip1^ proteins, but these effects were not visible at 20 µM. Effects of HMI-1a3 were completely different from PMA and bryostatin 1. Both of these reference compounds down-regulated cyclin A and p27, had no effect on cyclin B1, total Rb and pRb, strongly up-regulated cyclin D1 and p21, and slightly up-regulated cyclin E (Fig. 7A and 7B).

## Discussion

In efforts to develop drugs to treat PKC-related diseases, targeting the C1 domain has some advantages over the traditional approach of targeting the catalytic site. For example, it provides a possibility of developing activators. It also decreases the number of possible target proteins thus increasing chances for selectivity. Moreover, in addition to its catalytic activity PKC has noteworthy non-catalytic functions [15]. For instance, it has been shown that the pro-proliferative and pro-survival role of PKCα in several glioma cell lines is not dependent on kinase activity, but is rather dependent on the protein itself as a non-catalytic scaffold protein [16]. Furthermore, a conserved sequence N-terminal of the C1b domain of PKCε has been shown to be responsible for the induction of neurite-like processes in neuroblastoma cells [17], [18]. We have previously described properties of a novel group of C1 domain ligands, dialkyl 5-(hydroxymethyl)isophthalates [14]. In the present study, we showed that effects of selected isophthalates on HeLa cell viability and proliferation correlated well with the previously developed structure-activity model that was based on binding affinities. Compounds with good binding affinity to C1 domains of PKCα and PKCδ inhibited HeLa cell proliferation and induced cytotoxicity, while derivatives with poor binding affinity had no effect. Therefore, effects are probably mediated by either PKC or some other protein containing a DAG/phorbol-responsive C1 domain. Despite relatively similar binding affinities to PKCs, active compounds affected HeLa cell viability and proliferation at different potencies. For example, HMI-1a3 and HMI-1b11 bind to PKCα and PKCδ with comparable affinities [14], but HMI-1b11 exerts significantly less toxicity to HeLa cells. This observation is consistent with our previous studies showing that HMI-1a3 induced ERK1/2 activation in a PKC-dependent manner, and HMI-1b11 alone was unable to induce ERK1/2 phosphorylation but rather inhibited PMA-evoked ERK1/2 phosphorylation [14]. This difference between the compounds could be due to different outcomes associated with binding to the target protein (activation or inhibition) or different binding affinities to the target protein if the effect is mediated by a non-PKC phorbol ester receptor.

Natural C1 domain ligands PMA and bryostatin 1 that were used as reference compounds had no or only weak effects on HeLa cell viability. PMA-induced decrease in cell viability was statistically significant only at 10 nM concentration, and there was no clear antiproliferative effect at concentrations used in this study (1 nM-1 µM). Depending on the cell type, PMA may have opposing effects on cell proliferation and apoptosis [19]. Bryostatin 1 on the other hand has been reported to have little effect on HeLa cell proliferation [20], which was supported by our data from cell viability assays, and therefore it was not included in Cell-IQ® experiments.

Results from cell viability studies and Cell-IQ® experiments also indicate that the mechanism of isophthalate-induced cytotoxicity is non-necrotic because cytotoxic effects were observed only in the MTT assay and not the LDH assay, which measures cell membrane integrity [21]. Additionally, the mechanism of isophthalate-induced cell death seems to be caspase-independent because the pan-caspase inhibitor BAF had no effect on the kinetics of HeLa cell death in Cell-IQ® studies. Generally, caspase inhibitors decelerate the process of apoptotic cell death even though they are not able to prevent it [22], [23].

Thymidine incorporation assays with HMI-1a3 showed that the onset of the effect was rapid. Specifically, even a 6-h exposure to HMI-1a3 inhibited thymidine incorporation, although not as efficiently as a 24-h exposure. HMI-1a3-induced inhibition of thymidine incorporation at concentrations of ≥4 µM was consistent with the results from Cell-IQ® analysis. However, the increase in thymidine incorporation observed at the lowest HMI-1a3 and PMA concentrations was contrasted with results from Cell-IQ® time-lapse analysis. Specifically, the lowest concentrations of PMA and HMI-1a3 did not significantly increase cell proliferation at any time point upon analysis by Cell-IQ®. Thus, increases in thymidine incorporation do not necessarily reflect actual increases in cell proliferation. Furthermore, inhibition of thymidine incorporation at PMA concentrations of ≥100 nM were not supported by results from Cell-IQ® analysis, where PMA exposure was shown to have no prominent effect on HeLa cell proliferation.

While studying proliferation of HeLa cells with Cell-IQ®, we observed that compounds that inhibited cell proliferation also induced cell elongation. These cell elongations were characterized by narrowed cell bodies, elongated cell processes, and reduced cell-to-cell contacts. Similar changes in HeLa cell morphology have been recently reported as a consequence of knock-down of prohibitin [24], which plays important roles in mitochondrial function and regulating PI3K/Akt, Ras/ERK and TGF-β signaling pathways [25]. Elongated HeLa cell morphology has also been reported after chronic exposure to increasing concentrations of DMSO (0.5–2%) [26]. This study also used DMSO as the co-solvent for all compounds tested. However, DMSO concentrations were always ≤0.4% and had no effect on cell morphology or proliferation. Regarding C1 domain-containing proteins, PKCs, DGKs, chimaerins and MRCKs have been shown to participate in controlling cytoskeleton reorganization [27], [28] and thus may be responsible for mediating changes in cell morphology induced by isophthalates. The fact that PMA also induced cell elongation supports the hypothesis that the morphological changes are mediated by a DAG effector. However, PMA-induced cell elongation was transient and it was accompanied with emergence of rounded cells, a phenomenon not seen in isophthalate-treated cells. This may be due to different outcome (activation/inactivation/down-regulation) of ligand binding to its target. For example, C1 domain ligands are known to induce different patterns of PKC down-regulation [29], [30]. Phorbol esters and bryostatin 1 induce permanent activation of PKC isoforms, which eventually leads to their down-regulation and thus inhibition of kinase activity. Transient (such as DAG-induced) PKC activation on the other hand does not induce down-regulation. Furthermore, the existence of several classes of phorbol/DAG-responsive C1 domain-containing proteins and the cross-talk of their signaling pathways adds an additional layer of complexity to this issue.

Observations of higher proportions of elongated and non-dividing cells with longer exposure times to HMI-1a3 suggest that HMI-1a3 may exert its effects at a certain phase of the cell cycle, so that only cells in a specific cell cycle phase might be affected by treatments. This idea is supported by results showing that a 6-h treatment with HMI-1a3 was unable to completely block thymidine incorporation, suggesting that HMI-1a3 may not be able to inhibit ongoing DNA synthesis. Rather, it may induce cell cycle arrest just before cells enter the S phase. Therefore, we also studied effects of HMI-1a3 on the expression of several proteins related to cell cycle progression (reviewed in [31], [32]). Typically, D-type cyclins are expressed throughout the cell cycle, while cyclins A, B and E are expressed in a periodical manner: cyclin A is expressed in S and G2 phases of the cell cycle; cyclin B is expressed in S, G2 and M phases; and cyclin E expression is induced in late G1 phase. During G1/S transition Rb is inactivated by phosphorylation, leading to cell cycle progression. The cyclin-dependent kinase inhibitors p21 and p27 typically induce cell cycle arrest in G1 phase. Exposure to HMI-1a3 induced down-regulation of cyclins A, B1, D1, and E, suggesting that the proportion of cells in S, G2, M, and late G1 phases were reduced. Furthermore, HMI-1a3 exposure led to a pronounced down-regulation of Rb, which is generally regulated by phosphorylation/dephosphorylation and not by protein expression levels. Down-regulation of Rb protein has been reported previously in several cancer cell lines after exposures to an Mdm2 antagonist nutlin-3 [33]. While down-regulation of Rb would be expected to release E2F transcription factors and promote cell cycle progression, nutlin-3-induced down-regulation of Rb resulted in growth arrest or apoptosis, depending on the cell type. The effect was dependent on p53, linked to up-regulation of p21, and independent of the E2F transcription factor. Our results with HMI-1a3 are partially in agreement with these results; however, we did not observe up-regulation of p21. Aberrant cell cycle regulation in cervical cancer cell lines may explain the difference [34]. In human papilloma virus (HPV)-positive cervical cancer cells, such as HeLa cells, HPV E6 oncoprotein selectively targets p53 for degradation via ubiquitin-protein ligase E6-AP instead of Mdm2 [35], which is the main mediator of p53 degradation under normal growth conditions. The tumor suppressor protein p53 on the other hand regulates transcription of p21 [32], and thus the instability of p53 in HeLa cells may explain why up-regulation of p21 was not detected. Therefore, signaling pathways related to HMI-1a3-induced down-regulation of Rb and cell cycle arrest in HeLa cells are expected to be somewhat different than those related to nutlin-3. Nevertheless, HMI-1a3-induced down-regulation of cyclins provides evidence supporting the hypothesis that the cell cycle may arrest in G1 phase before induction of cyclin E expression.

The results of this study substantiate the potential of C1 domain ligands in drug discovery. Targeting the regulatory domain of PKC may be more beneficial than targeting the catalytic site in the development of anticancer therapeutics. C1 domain-targeted PKC activators may also provide potential therapeutic treatments for Alzheimer's disease [36]. Additionally, non-PKC DAG effectors and the complicated signaling networks in which C1 domain-containing proteins interact with each other provide numerous opportunities for therapeutic discoveries. Furthermore, the results presented in this article point out the potential of a novel group of C1 domain ligands, dialkyl *5*-(hydroxymethyl)isophthalates, as antiproliferative agents. Although the binding affinities of isophthalates for PKC isoenzymes are lower than those reported for some other classes of synthetic C1 domain ligands such as DAG lactones and benzolactams, cellular effects seem to appear at similar concentrations. For example, a benzolactam derivative has been reported to inhibit breast cancer cell proliferation with IC_50_ values of 20–30 µM depending on the cell line [37], and induction of apoptosis by DAG lactones has been reported using concentrations of 10–20 µM [38], [39]. Natural products such as ingenol-3-angelate and bryostatin 1 are notably more potent and exert their cellular effects at nanomolar concentrations [12], although bryostatin 1 alone has little antiproliferative or proapoptotic efficacy [40]. Structural complexity of these naturally occurring compounds however hinders their use in the development of improved derivatives and therefore simpler templates are acutely needed.

C1 domain-targeted small molecules present promising potential as drug candidates for the treatment of cancer and Alzheimer's disease. This study is the first report of cellular activity of a novel group of C1 domain ligands, dialkyl 5-(hydroxymethyl)isophthalates. We show here that active isophthalate derivatives induced cell cycle arrest and non-necrotic, caspase-independent cell death in HeLa human cervical cancer cells. The most potent isophthalate derivative, HMI-1a3, exerted its effect at low micromolar concentrations comparable to those reported with other established synthetic C1 domain ligands. However, the considerably simpler synthesis route makes the isophthalate derivatives especially attractive candidates for further development as lead compounds in drug development. In contrast, bryostatin 1 and PMA had no major effects of HeLa cell viability and proliferation, and therefore their effects on the expression of cell cycle markers were also distinct from those induced by HMI-1a3. Even though the exact mechanism of action by which isophthalates induce their effects in HeLa cells is for the present under investigation, the results strongly indicate that the effect is C1 domain-mediated.

## Materials and Methods

### Materials

All reagents and chemicals were commercially available, except for isophthalic acid derivatives that were designed and synthesized at the Division of Pharmaceutical Chemistry, Faculty of Pharmacy, University of Helsinki (Finland) [14]. Phorbol 12-myristate-13-acetate (PMA) and bryostatin 1 were purchased from Sigma-Aldrich (Steinheim, Germany). Cell culture solutions and reagents were purchased from Invitrogen (Carlsbad, CA, USA) except for Dulbecco's modified Eagle's medium (DMEM), which was purchased from Sigma-Aldrich (Steinheim, Germany). The reagent [methyl-^3^H] thymidine was purchased from Amersham Pharmacia Biotech (Buckinghamshire, UK). Primary antibodies against p21^waf1/cip1^, p27^kip1^, cyclin A, cyclin B1, cyclin D1, cyclin E, retinoblastoma (Rb), phosphorylated form of Rb (Ser-795; pRb) and β-actin were obtained from Cell Signaling Technology (Danvers, MA, USA) as were HRP-linked secondary antibodies anti-mouse IgG and anti-rabbit IgG. Anti-GAPDH monoclonal antibody was purchased from Santa Cruz Biotechnology Inc. (Santa Cruz, CA, USA). Precision Plus Protein Kaleidoscope standard ladder (Bio-Rad Laboratories, Hercules, CA, USA) was used as the sodium dodecyl sulfate polyacrylamide gel electrophoresis (SDS-PAGE) marker. Bicinchoninic acid (BCA) protein assay reagents (BCA Protein Assay Kit) and enhanced chemiluminescence (ECL) reagents (SuperSignal-West-Pico-Chemiluminescent-Substrate-Kit) were purchased from Pierce (Thermo Fisher Scientific Inc., Rockford, IL, USA). The pan-caspase inhibitor Boc-Asp(OMe) fluoromethyl ketone (BAF) was purchased from Sigma-Aldrich (Steinheim, Germany).

### Cell culture

HeLa cells (CCL-2) were acquired from American Type Culture Collection (ATCC, Manassas, VA, USA) and cultured in DMEM supplemented with 10% fetal bovine serum (FBS), 100 U/mL of penicillin, and 100* µ*g/mL of streptomycin. The same medium was used for all treatments. Cultures were incubated at 37°C in a humidified atmosphere of 5% carbon dioxide (CO_2_).

### Cell viability tests

Cells were seeded onto 96-well plates (10^4^ cells/well) and incubated overnight to allow attachment. Cells were then treated with test compounds in serum-supplemented DMEM (150 µL/well) in a humidified, 5%-CO_2_ atmosphere at 37°C for 24 or 48 h after which standard mitochondrial dehydrogenase activity (MTT) and lactate dehydrogenase activity (LDH) tests were performed. **For LDH tests,** 50 µL of medium from each well was transferred into a new 96-well plate followed by addition of 50 µL of substrate solution (1.3 mM β-nicotinamide adenine dinucleotide (β-NAD), 660 µM iodonitrotetrazolium, 54 mM L(+)-lactic acid, 280 µM phenazine methosulphate, 0.2 M Tris-HCl (pH 8.2). Plates were gently shaken for 10 min and incubated for another 20 min at room temperature (RT). Fifty microliters of acetic acid (1 M) was added to stop the reaction, and absorbance was measured at 490 nm (1 s/well). Background absorbance was measured in wells containing medium alone. Spontaneous LDH release was measured from untreated cells, and maximal LDH release was determined from cells treated with 9% Triton X-100. **For MTT tests,** 3-(4,5-dimethylthiazol-2-yl)-2,5-diphenyltetrazolium bromide (MTT) was added to cells yielding a final concentration of 0.5 mg/mL, and plates were incubated for 2 h at 37°C in a 5%-CO_2_ atmosphere. The medium was then aspirated and 200 µL of DMSO was added to each well. Absorbance was measured at 550 nm with absorbance at 650 nm extracted as background.

### Thymidine incorporation

HeLa cells were seeded onto 6-well plates (4×10^5^ cells/well) and incubated overnight to allow cells to attach. Cells were treated with compounds or vehicle, and 0.5 µCi/mL [methyl-^3^H]thymidine was added to cells for the last 6 h of the incubation. After the incubation, cells were washed three times with cold phosphate-buffered saline (PBS). Ice-cold trichloroacetic acid (5% TCA) was added and plates were incubated on ice for 10 min to extract residual thymidine. The TCA solution was discarded, cells were lysed with 0.1 M NaOH and radioactivity was measured.

### Cell proliferation and morphology studies

HeLa cells were seeded onto 48-well plates (5000 cells/well) and left to attach overnight. Cells were treated with compounds and grown on a continuous cell culturing platform with integrated optics for phase contrast imaging and machine vision technology (Cell-IQ®, Chip-man Technologies Ltd, Tampere, Finland) in a humidified, 5%-CO_2_ atmosphere at 37°C for 72 h. Images were captured automatically from 3–4 positions per well at 1 h intervals. A protocol for examining cell numbers and morphology of HeLa cells with Cell-IQ Analyzer® software was created according to manufacturer instructions. Briefly, segmentation parameters were first adjusted to achieve optimal cell recognition. Recognized cells were then classified into dead, dividing, elongated and healthy HeLa cells. In addition, a class was added for false recognitions of background. The protocol was tested several times and optimized by analyzing a set of sample images to verify classifications from images. The protocol was also tested for accuracy by comparing classifications from a set of sample images to classifications made manually by three researchers. The protocol was then used to analyze images taken during Cell-IQ® experiments. Data were analyzed in three steps using Microsoft Excel. First, false recognitions were subtracted from total cell numbers. Second, total cell numbers were normalized to total cell numbers in the same position at the beginning of the experiment (i.e., the first image from each position). Finally, numbers of dead, dividing, elongated and healthy cells were converted to percentage of total cell number (false recognitions subtracted).

### Determination of cell cycle markers by Western blotting

HeLa cells were seeded onto 6-well plates (4×10^5^ cells/well) and left overnight to attach. Cells were treated with test compounds for 24 h, washed three times with PBS and lysed with 1% SDS in 1 mM of Tris-HCl (pH 7.0). The lysates were sonicated and centrifuged at 16000 *g* for 15 min at 4°C. Pellets were discarded and the protein concentration of the supernatant was measured using a BCA protein assay kit according to manufacturer's instructions. Samples were diluted in Laemmli sample buffer at equal concentrations and stored at −20°C until use. Twenty micrograms of protein per lane were subjected to SDS-PAGE and subsequently transferred to nitrocellulose membranes. Membranes were washed for 5 min with 0.1% Tween 20 in Tris-buffered saline (TTBS) and then blocked for 1 h with 5% nonfat milk powder in TTBS (milk-TTBS) at RT. Membranes were incubated at 4°C overnight with the primary antibody in milk-TTBS after which they were washed for 35 min with TTBS. Membranes were then incubated with horseradish peroxidase-conjugated secondary antibody (goat anti-rabbit IgG or goat anti-mouse IgG, 1∶2000 in milk-TTBS) for 1 h at RT. After washing for 40 min with TTBS, bands were visualized with ECL. GAPDH and β-actin bands were used as controls to ensure equal protein loads among all wells. Western blots were quantified by measuring the optical density of the immunoreactive bands with Scion Image software (http://scioncorp.com).

### Statistical analysis

Data are expressed as the mean ± SEM from at least three independent experiments unless otherwise stated. MTT and Cell-IQ® results were analyzed using PASW Statistics 18 software (SPSS Inc., Chicago, IL, USA). Concentration-response data were analyzed using one-way analysis of variance (ANOVA) followed by a Tukey's honestly significant different (HSD) post hoc test.
