# Correction: C1 Domain-Targeted Isophthalate Derivatives Induce Cell Elongation and Cell Cycle Arrest in HeLa Cells

**DOI:** 10.1371/annotation/7608bd2a-e319-47fd-ae20-478ee2875e27

**Published:** 2011-06-30

**Authors:** Virpi Talman, Raimo K. Tuominen, Gustav Boije af Gennäs, Jari Yli-Kauhaluoma, Elina Ekokoski

The third author's name appears incorrectly in the citation. The correct citation is: Talman V, Tuominen RK, Boije af Gennäs G, Yli-Kauhaluoma J, Ekokoski E (2011) C1 Domain-Targeted Isophthalate Derivatives Induce Cell Elongation and Cell Cycle Arrest in HeLa Cells. PLoS ONE 6(5): e20053. doi:10.1371/journal.pone.0020053 

